# Deciphering the Molecular Mechanism of the Intermediate Secondary Growth and Internode Elongation of the Castor Bean (*Ricinus communis* L.) by the Combined Analysis of the Transcriptome and Metabolome

**DOI:** 10.3390/ijms25021053

**Published:** 2024-01-15

**Authors:** Yujie Chen, Yuriy L. Orlov, Ming Chen

**Affiliations:** 1 College of Life Sciences, Zhejiang University, Hangzhou 310058, China; chenyujie@imun.edu.cn; 2 College of Life Sciences and Food Engineering, Inner Mongolia Minzu University, Tongliao 028000, China; 3 Agrarian and Technological Institute, Peoples’ Friendship University of Russia, 117198 Moscow, Russia; orlov@d-health.institute

**Keywords:** IAA, internode development, lignification, main stem, plant height, secondary cell wall

## Abstract

The length of internodes plays a crucial role in determining the height of the castor plant (*Ricinus communis* L.). However, the specific mechanisms underlying internode elongation, particularly in the main stem of the castor plant, remain uncertain. To further investigate this, we conducted a study focusing on the internode tissue of the dwarf castor variety 071113, comparing it with the control high-stalk Zhuansihao. Our study included a cytological observation, physiological measurement, transcriptome sequencing, and metabolic determination. Our integrated findings reveal that the dwarf variety 071113 undergoes an earlier lignification development in the main stem and has a more active lignin synthesis pathway during internode intermediate development. In addition, the dwarf variety exhibited lower levels of the plant hormone indole-3-acetic acid (IAA), which had an impact on the development process. Furthermore, we identified specific enzymes and regulators that were enriched in the pathways of the cell cycle, auxin signal transduction, and secondary cell wall synthesis. Using these findings, we developed a model that explained the intermediate secondary growth observed in castor internode elongation and enhanced our comprehension of the dwarfing mechanism of the 071113 variety. This research provides a theoretical groundwork for the future breeding of dwarf castor varieties.

## 1. Introduction

Plant architecture is a crucial characteristic in agriculture, particularly for crops with compact and dwarf structures. These types of plants offer several advantages, including the potential to increase planting density and yield, as well as strong resistance to stalk lodging and pathogen infection. Furthermore, compact and dwarf plants are well-suited to mechanized management and harvesting [1,2]. The promotion of dwarf breeding during the “Green Revolution” in the 1960s played a critical role in enhancing crop yield [3]. The height of a plant is determined by the number of nodes and the length of internodes. Internode elongation is a complex process influenced by various factors, such as hormones, genetic factors, and the development of the secondary cell wall in internodes [4,5].

Numerous plant hormones, including indole-3-acetic acid (IAA), gibberellins (GAs), brassinosteroids (BRs), abscisic acid (ABA), and cytokinin (CTK), function as key regulators of internode elongation development [6,7]. In the IAA pathway, transcription factors, such as auxin response factors (ARFs), play a crucial role in regulating internodal elongation by activating expansin (EXP) proteins involved in cell wall synthesis [8]. Furthermore, the growth of the stem’s vascular tissue, culminating in the formation of xylem cells with a secondary wall, is paramount for effective water transportation and a taller stature in plants, which consequently affects the plant’s height [9]. The characteristic morphology of cells, tissues, organs, and holistic plant structure owes its definition to the cell walls that encase plant cells [10,11,12]. However, transcriptome experiments on entire internodes of nettle (*Urtica dioica* L.) highlight a distinctive transcriptomic profile for the elongating and mature nettle internode. The development of secondary walls typically hinders cell growth, thereby restricting internode elongation [13]. Additionally, plant height is notably influenced by the biosynthesis of three primary components in the secondary cell wall, namely, cellulose, hemicellulose, and lignin, which regulate the plant’s stature [14].

The castor bean (*Ricinus communis* L.) is a valuable oil crop celebrated for its high oil content (50% to 70%), particularly castor oil, used extensively in biodiesel production and pharmaceutical industries due to its unique ricinoleic acid (12-hydroxyoleic acid, 18C:1OH). Its cultivation is predominantly in Africa and Asia, valued for its resilience to both drought and cold conditions, and its ability to flourish in marginal lands [15]. In China, the Tongliao region in Inner Mongolia holds significant importance for castor bean production. The plant’s height substantially determines its yield potential. Presently, most propagated varieties in China are high-stalk types, presenting certain drawbacks, including an extended growth period, broad crown width, and irregular maturity periods among lateral branches. Such characteristics inhibit mechanized management [16]. To maximize the yield potential of castor bean crops, it is imperative to develop dwarf varieties and enhance their population structure. 

Plant height in the castor bean crop, a typical quantitative trait, presents a positive correlation with the height of the primary raceme (PRH), the number of main stem nodes (MSNNs), and the length of the main stem internode (MSIL). Notably, this complex quantitative trait follows a normal distribution in the F_2_ generation, which reveals one or two Quantitative Trait Loci (QTLs) during the breeding process [17]. The main stem of the castor bean plant consists of nodes and internodes. Li Jinqin’s research [18] demonstrates that dwarf castor plants display a notable reduction in internode length and a more compact structure. Conversely, high-stalk castor plants do not exhibit these characteristics, suggesting that internode development significantly impacts plant height. 

Therefore, shortening the internodes of the main stem is a critical breakthrough in dwarf breeding. The development of plants can be roughly divided into the seedling stage, budding stage, flowering stage, flowering and fruiting stages, and filling and maturing stages. The height of castor plants undergoes rapid growth from budding to flowering, characterized by the fastest development of the MSIL. This period is critical for determining the final height of castor plants [19]. Feng conducted a research study [20] using dwarf castor 3358 and high-stalk castor 2129 to create a hybrid population of F_2_. A RNA-Seq comparison revealed a total of 6557 differentially expressed genes (DEGs) between the tall and dwarf castor plants in the F_2_ generation. A functional enrichment analysis demonstrated that these genes were significantly enriched in the plant hormone signal transduction pathway. Lu found that RcPAL was a vital enzyme in lignin biosynthesis in castor plants. The *RcPAL* gene is triggered by mechanical damage stress and its increased expression results in a higher lignin content and reduced plant height [16]. 

The internode elongation of the castor plant is a crucial factor for determining its overall height. There have been some observations of a correlation between secondary growth, hormone regulation, and plant height. However, the specific molecular mechanism underlying this relationship remains unknown. Understanding this mechanism is of utmost importance for castor breeding purposes. Therefore, our research aims to investigate the correlation between secondary growth and plant height in the intermediate tissues of the main stem of dwarf castor plants, with Zhuansihao as the control. The objective is to elucidate the molecular development mechanism of the 071113 dwarf castor variety by comparing the cytological characteristics, physiological characteristics, hormone levels, transcriptome, and metabolic differences in the intermediate tissues of the main stem of dwarf castor plants.

## 2. Results

### 2.1. Data Associated with Plant Height

**Figure 1:**
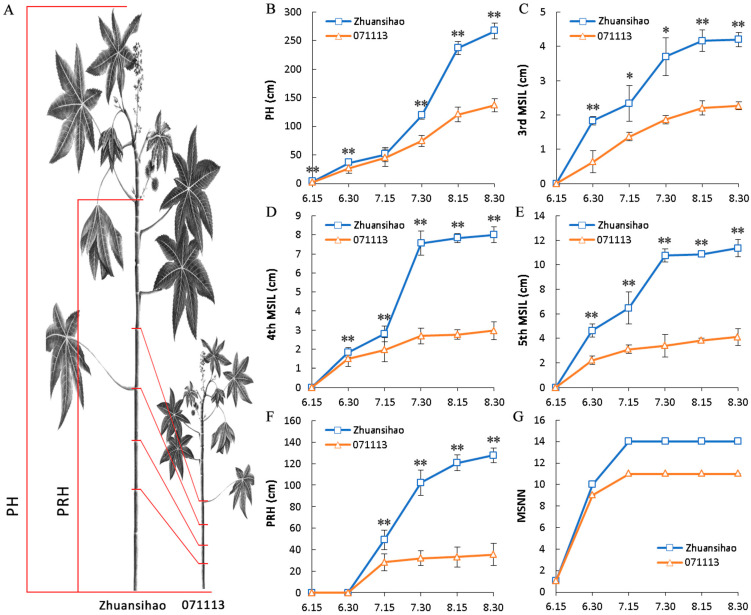
Plant heights of high-stalk and dwarf castor plants at various stages of development, with Zhuansihao serving as the control and 071113 as the experimental group. (**A**) Schematic diagram of high-stalk and dwarf castor plants. (**B**) PH. (**C**) The 3rd MSIL. (**D**) The 4th MSIL. (**E**) The 5th MSIL. (**F**) The PRH. (**G**) The MSNN. Note: PH: plant height; PRH: the height of the primary raceme; MSNNs: the number of main stem nodes; MSIL: the length of the main stem internode. “*” Significant difference, “**” Very significant difference.

The phenotypic data from two commonly cultivated varieties in the Tongliao region in Inner Mongolia, namely, short-stalk 071113 and high-stalk Zhuansihao, were collected and compared. The average heights of grown plants for Zhuansihao and 071113 were 266.9 ± 6.9 cm and 136.8 ± 5.3 cm, respectively (Figure 1A,B). The plant heights, mainly determined by the MSIL of the third, fourth, and fifth main stem nodes, exhibited significant differences. During the rapid growth period of the castor plant (from mid-July to mid-August), the Zhuansihao plant height increased by almost 200 cm, while short-stalk 071113 only increased by about 50 cm (Figure 1C–E). Zhuansihao demonstrated a faster growth rate compared to 071113, with the largest difference in plant height observed between the two varieties during this period. The PRH of high-stalk castor plants began to differ from the end of June and continued to increase until they stopped growing in mid-August. Conversely, the PRH of dwarf castor ceased growth in mid-July, and the MSNN difference between the two was not significant (Figure 1F,G). Therefore, the length of the fifth internode of the castor main stem exhibited the most significant difference between the two varieties.

### 2.2. Examination of Cellular Characteristics

**Figure 2:**
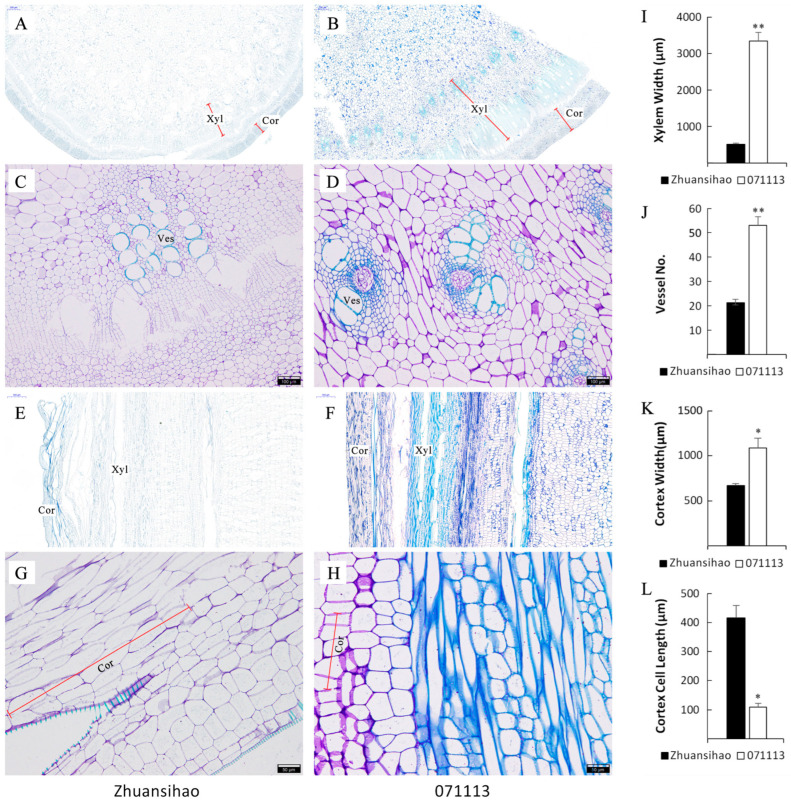
The histological and cytological differences between high-stalk and dwarf castor plant 5th internodes, with Zhuansihao serving as the control and 071113 as the experimental group. (**A**) Cross-section of the Zhuansihao stem; scale bar = 500 μm. (**B**) Cross-section of 071113 stem; scale bar = 500 μm. (**C**) Partial zoom of Zhuansihao stem cross-section; scale bar = 100 μm. (**D**) Partial zoom of 071113 stem cross-section; scale bar = 100 μm. (**E**) Longitudinal section of Zhuansihao stem; scale bar = 500 μm. (**F**) Longitudinal section of 071113 stem; scale bar = 500 μm. (**G**) Partial zoom of Zhuansihao longitudinal section; scale bar = 50 μm. (**H**) Partial zoom of 071113 longitudinal section; scale bar = 50 μm. (**I**) Xylem width. (**J**) Vessel number. (**K**) Cortex width. (**L**) Cortex cell length. Note: Xyl: xylem; Cor: cortex; Ves: vessel. * indicates a difference at *p* value < 0.05, ** indicates a difference at *p* value < 0.01.

The paraffin-embedded sections of the fifth internode tissues of two varieties of dwarf castor plants, namely, short-stalk 071113 and high-stalk Zhuansihao, were compared during the budding stage of growth. The use of toluidine blue dye facilitated the visualization of lignified structures as blue and non-lignified structures as purple. The cross-section of the internode in both castor varieties exhibited typical characteristics of a dicotyledonous plant stem, including an epidermis, cortex, and vascular column structure comprising vascular bundles, pith, and pith rays. A distinct bundle-like structure consisting of metaxylem was observed in the dwarf castor, which included both xylem and phloem (Figure 2A,B). In dwarf castor 071113, the cell wall of vessel cells and surrounding parenchyma cells showed lignification with blue staining, while Zhuansihao exhibited blue staining only in a portion of the vessel cells. Dwarf castor 071113 displayed a more developed vascular system and secondary structures in its internode tissue compared to the high-stalk variety (Figure 2C,D). Furthermore, dwarf 071113 had a larger cortex width (Figure 2E,F) and a shorter length of cortical cells (Figure 2G–L).

### 2.3. Content Determinations of Phytohormone, Lignin, and Cellulose

**Figure 3:**
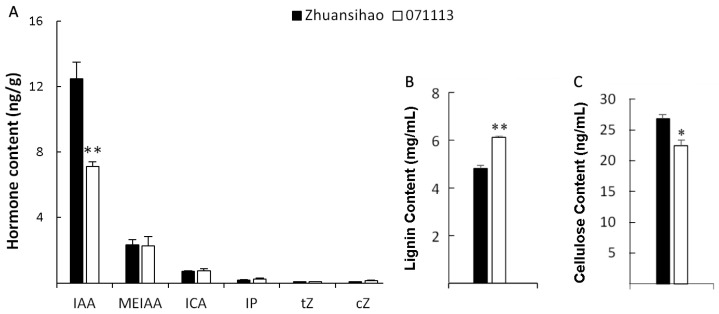
Comparison of the contents of hormone, lignin, and cellulose between high-stalk and dwarf castor plant internodes, with Zhuansihao serving as the control and 071113 as the experimental group. (**A**) Hormone contents. (**B**) Lignin contents. (**C**) Cellulose contents. Note: IAA: indole-3-acetic acid; MEIAA: methyl indole-3-acetate (MEIAA); ICA: indole-3-carboxaldehyde; IP: N6-Isopentenyladenine; tZ: trans-Zeatin; cZ: cis-Zeatin. * indicates a difference at *p* value < 0.05, ** indicates a difference at *p* value < 0.01.

The study identified three types of auxin hormones, namely, indole-3-acetic acid (IAA), methyl indole-3-acetate (MEIAA), and indole-3-carboxaldehyde (ICA), as well as three types of cytokinin hormones, N6-Isopentenyladenine (IP), trans-Zeatin (tZ), and cis-Zeatin (cZ), in both short-stalk and high-stalk castor internodes. Notably, the concentration of IAA was significantly higher compared to the levels of the other hormones. These findings indicate that IAA plays a crucial role in regulating the growth of internode tissue in castor stems. Furthermore, when comparing the different types of auxins and cytokinins analyzed, the study observed significantly lower levels of IAA in the fifth internode tissue of dwarf castor plants compared to tall castor plants. This difference was found to be statistically significant (Figure 3A).

The lignin and cellulose contents in the internode tissue were measured, and it was found that short-stalk castor had a higher average lignin content (6.12 ± 0.58 mg/mL) compared to high-stalk castor (4.82 ± 0.23 mg/mL). The cellulose content in dwarf castor (22.42 ± 1.67 ng/mL) was significantly lower than that in high-stalk castor (26.80 ± 1.25 ng/mL), indicating an inverse relationship to the lignin content (Figure 3B,C). In the fifth internode of the castor stem, the lignin content was much higher than the cellulose content. Furthermore, the lignin content of castor 071113 was much higher than that of castor Zhuansihao, suggesting that the intermediate secondary growth of short-stalk castor was more developed than that of high-stalk castor at this development stage.

### 2.4. Metabolome Analysis

The principal component analysis (PCA) of the metabolome clearly differentiated between the high-stalk group (Group L) and the short-stalk group (Group D), each group consisting of three biological replicates. The Pearson correlation coefficient (PCC) values between the biological replicates indicated strong relationships, with all r^2^ values exceeding 0.88. A total of 621 castor metabolites were detected in both groups, which were classified into 11 different categories. These categories included primary metabolites, like lipids, organic acids, sugars, nucleotides, amino acids and their derivatives, as well as secondary metabolites, such as phenylpropanoids, alkaloids, terpenes, tannins, steroids, and quinones. Based on the criteria of a fold change (FC) value greater than or equal to 1.5 or less than or equal to 0.5 of the D/L ratios, and a variable importance in project (VIP) value of at least 1, a total of 42 differential metabolites (DMs) were identified. Among these DMs, 26 were up-regulated and 16 were down-regulated (Table 1). The KEGG pathway enrichment analysis indicated that the 42 DMs mainly belonged to the pathways of biosynthesis of various secondary metabolites, phenylpropanoid biosynthesis, and flavonoid biosynthesis. 

**Figure 4:**
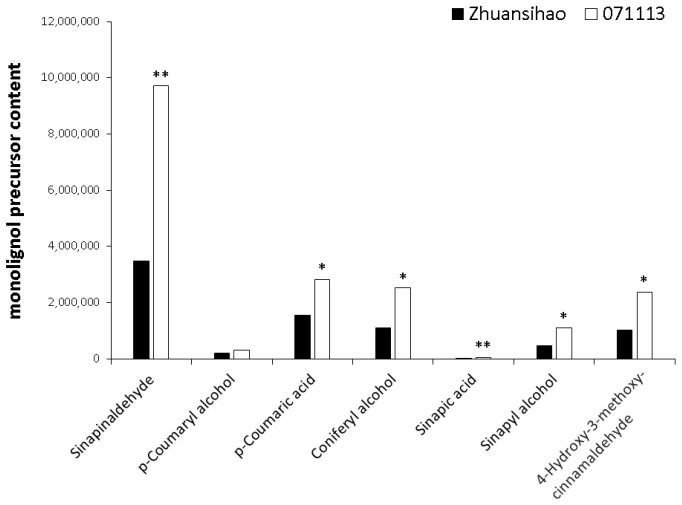
Comparison of the contents of lignin precursors and oligosaccharides between high-stalk and dwarf castor plant internodes, with Zhuansihao serving as the control and 071113 as the experimental group. Lignin precursor contents. * indicates a difference at *p* value < 0.05, ** indicates a difference at *p* value < 0.01.

Macromolecular lignin is primarily formed through the polymerization of three small molecular monomers: coniferyl alcohol (G monomer), sinapyl alcohol (S monomer), and p-coumaryl alcohol (H monomer). A metabolomic analysis revealed that, in the fifth internode tissue of the dwarf castor plant, the contents of G and S monomers were higher when compared to tall castor. Furthermore, a metabolome comparison detected the presence of various precursors, such as sinapic acid, sinapinaldehyde, p-coumaric acid, and 4-hydroxy-3-methoxycinnamaldehyde, in both varieties. The contents of these precursors were higher in dwarf castor than in tall castor (Figure 4). 

### 2.5. Transcriptome Sequencing

Clean reads were obtained after filtering the RNA-Seq sequencing data. The sequencing quality, with an average GC content of 42.73% and an average Q30 value of 92.43%, was suitable for further analysis. The PCC values of samples within and between groups resulted in the removal of the D2 sample. The D1 and D3 samples were considered biological replicates of the Dgroup samples. The L group consisted of L1, L2, and L3 samples, which were analyzed as biological replicates. Subsequently, screening was conducted to identify differentially expressed genes (DEGs) where the screening conditions were D/L |log2Fold Change| ≥ 2 and FDR < 0.05. A total of 1288 DEGs were identified, with 523 up-regulated genes and 765 down-regulated genes in dwarf castor. 

**Figure 5:**
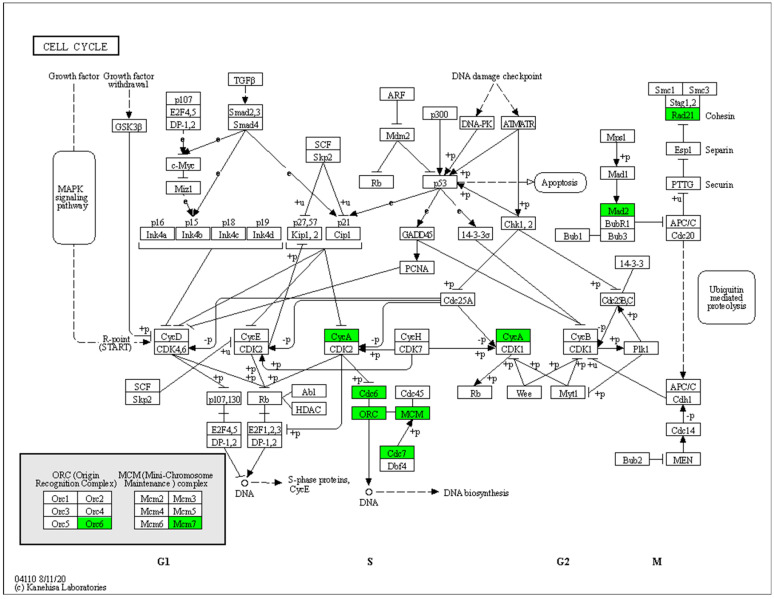
Cell cycle metabolic pathway, with Zhuansihao serving as the control and 071113 as the experimental group. The downregulated genes identified through RNA-Seq sequencing in the 071113 experimental group are shown in green.

**Figure 6:**
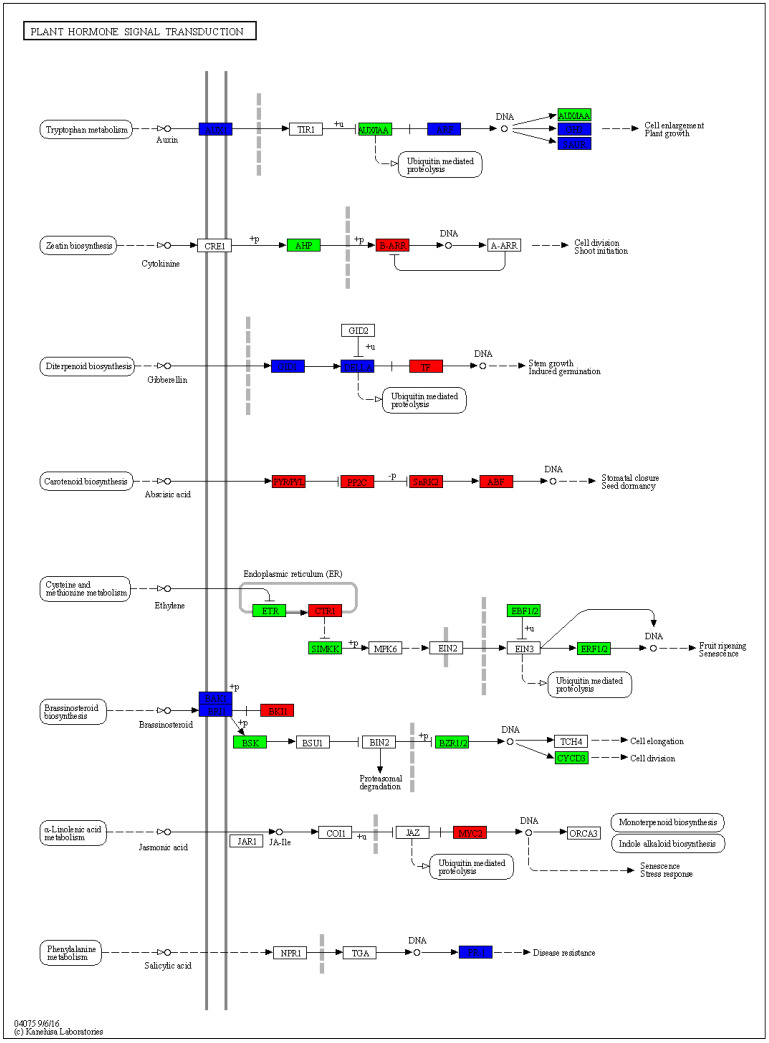
Plant hormone signal transduction pathway, with Zhuansihao serving as the control and 071113 as the experimental group. In this figure, up-regulated genes are depicted in red, while down-regulated genes are shown in green. Both up-regulated and down-regulated genes are shown in blue. These genes were identified through RNA-Seq sequencing in the 071113 experimental group.

The KEGG enrichment analysis indicated that several DEGs were predominantly associated with metabolic pathways involved in cell division. These pathways included the cell cycle, DNA replication, purine biosynthesis, and pyrimidine biosynthesis. Within the cell cycle pathway, a total of eight DEGs were down-regulated in short-stalk castor (Figure 5). *Cohesin complex subunit Rad21* (*LOC8261128*) and *mitotic spindle assembly checkpoint protein Mad2* (*LOC8278426*, *LOC8272625*) were involved in the spindle assembly checkpoint signaling pathway. Additionally, *cyclin-A* (*LOC8288529*), *the cell division control protein* (*LOC8283237*), and *the origin recognition complex subunit* (*LOC8279589*, *LOC8260621*, *LOC8274440*) were also part of this pathway. Furthermore, certain DEGs were found to be enriched in the plant hormone signal transduction pathway. In the auxin signal transduction pathway (Figure 6), several genes, such as *auxin efflux carrier component 3* (*LOC8258189*), *auxin-induced protein AUX22* (*LOC8271341*), and *ARF* (*LOC8259066*, *LOC8274635*) were down-regulated in the short-stalk 071113 variety, while only a few genes were up-regulated. In the high-stalk castor variety, *Histidine-containing phosphotransfer protein 4* (*LOC8285833*) was up-regulated in the cytokinin signal transduction pathway. On the other hand, all DEGs were up-regulated in the abscisic acid and jasmonic acid signal transduction pathway in the dwarf castor variety. In the lignin synthesis pathway, certain gene members of the peroxidase family were up-regulated, while others were down-regulated.. The signal transduction pathways of brassinosteroid, salicylic acid, and ethylene exhibited both up-regulated and down-regulated expressions. Moreover, the expressions of several members of the Expansin gene family, including *EXPB3* (*LOC8262087*), *EXLA2* (*LOC8278145*), and *EXPA4* (*LOC8282200*), were down-regulated in the dwarf plant.

### 2.6. Verification of Differentially Expressed Genes

**Figure 7:**
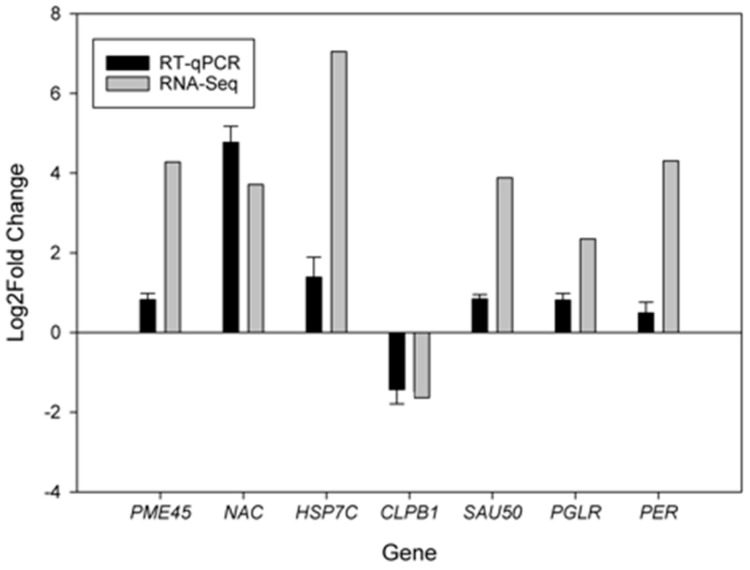
Verification of RNA-Seq sequencing data of DEGs for castor 071113 and Zhuansihao in internode tissues.

Seven DEGs, enriched in the pathways related to auxin signal transduction (*SAU50*, *LOC107260773*), cell wall biosynthesis (*PER*, *LOC8258115*; *PME45*, *LOC8288420*; *NAC*, *LOC8265938*; *PGLR*, *LOC107262217*), and protein processing in the endoplasmic reticulum (*HSP7C*, *LOC8265956*; *CLPB1*, *LOC8284441*) were selected and their expression patterns were verified by the quantitative polymerase chain reaction (real-time qPCR). The expression patterns of these seven genes were found to be consistent with the results obtained from the RNA sequencing analysis, indicating that the RNA-seq results were highly reliable (Figure 7). 

### 2.7. Metabolome and Transcriptome Correlation Analysis

**Figure 8:**
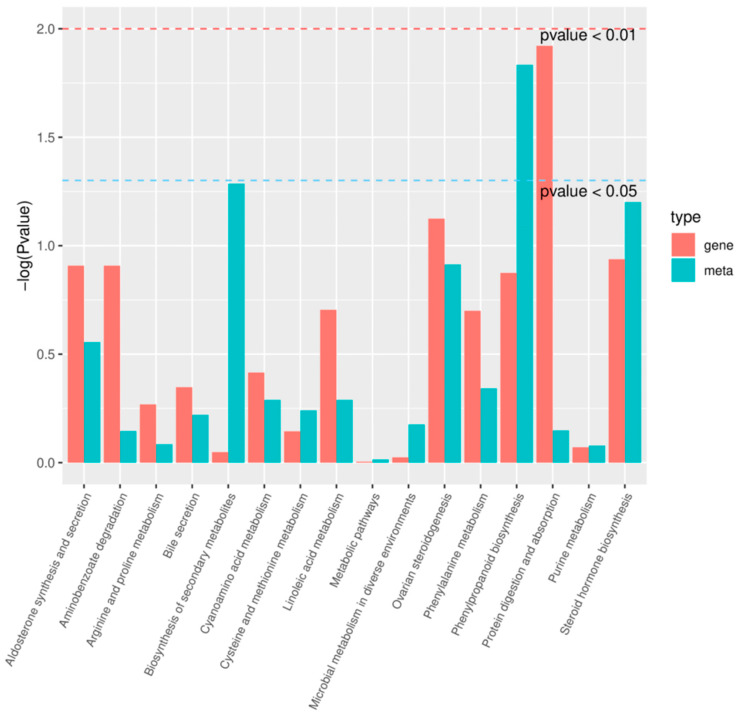
The correlation analysis between the metabolome and transcriptome. The blue dashed line indicates *p* value of less than 0.05, while the red dashed line indicates *p* value of less than 0.01.

**Figure 9:**
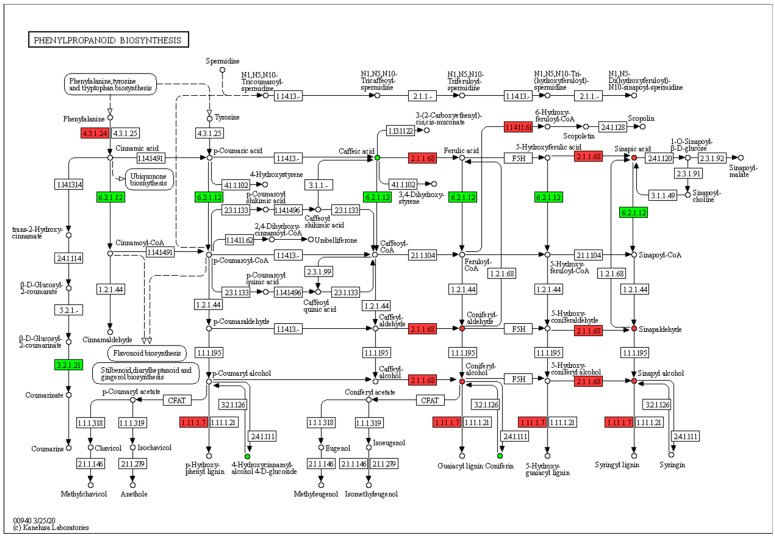
Phenylpropanoid biosynthesis pathway, with Zhuansihao serving as the control and 071113 as the experimental group. Three main precursor molecules of the lignin polymer are p-coumaroyl-CoA, coniferyl alcohol, and sinapyl alcohol. Among these, coniferyl alcohol and sinapyl alcohol have higher content levels in the dwarf 071113 variety compared to the high-stalk Zhuansihao variety. Additionally, two key enzyme encoding genes, namely Phenylalanine ammonia-lyase (4.3.1.24) and Peroxidase (1.11.1.7), show up-regulation in the dwarf 071113 variety compared to the high-stalk Zhuansihao variety.

The differentially expressed genes and differential metabolites were mapped to the KEGG pathway database to identify common pathways with the highest enrichment. Only those genes and metabolites with a *p* value less than 0.05 were considered for the analysis. The results indicate that the phenylpropane biosynthesis pathway exhibited the most significant enrichment in differential metabolites(Figure 8). Therefore, we conducted a detailed analysis of the expression patterns within the lignin synthesis pathway, considering both the metabolome and transcriptome. This pathway involves the synthesis of phenylpropanoids from phenylalanine, resulting in the production of coumaric acid and other acids. These acids are then converted into aldehydes and monolignols, which serve as the fundamental componentsof lignin. Phenylalanine ammonia-lyase (PAL) is the primary enzyme involved in the biosynthesis of lignin monomers. It is also the first enzyme in the phenylpropane metabolism pathway and acts as a rate-limiting step. Its main function is to facilitate the catalytic deamination of phenylalanine, generating trans-cinnamic acid, while peroxidase is the rate-limiting enzyme in the last step of lignin biosynthesis, which catalyzes the polymerization of lignin monomers into large molecules. In the internode tissue of dwarf castor beans, several key genes involved in lignin synthesis, such as *PAL* (*LOC8272514*, *LOC8272515*) and *Peroxidase* (*LOC8258115*, *LOC8271940*, *LOC8289058*), were up-regulated. Furthermore, the metabolome data support these findings as they show a higher abundance of lignin precursors in the dwarf castor compared to the tall castor (Figure 9).

## 3. Discussion

The length of internodes plays a critical role in determining the height of castor plants. Comparing the cellular structures of short-stalk and high-stalk castor varieties, it was found that short-stalk castor plants had shorter cortical cells in their internode tissues. Additionally, there are substantial differences in the number of xylem vessels between the two types of castor plants. Toluidine blue staining confirmed the presence of lignification in both types of plants. However, the dwarf castor variety 071113 exhibited intermediate secondary growth and a higher degree of lignification, while the tall castor plants had not yet initiated the development of the secondary cell wall. The exact cause of dwarfism in castor plants remains poorly understood with limited research. However, there are some potential factors that can contribute to dwarfism, such as the role of plant hormones and secondary cell wall lignification in regulating growth and development, specifically internode elongation. 

### 3.1. Auxin Is Required for Castor Internode Elongation

Imbalances in hormone levels or disrupted hormonal signaling pathways can impair internode growth and contribute to dwarfism [21]. In plants, hormones like auxin, GAs, and BRs are known to influence stem elongation. Any alterations in their biosynthesis or signaling pathways can potentially affect the development of internodes [22]. Auxin is widely recognized as a crucial regulator for controlling height due to its significant functions in regulating cell division, elongation, and differentiation [23]. In taller plants, auxin can be transported either through long-distance vascular transport or short-range active transport. The latter, known as auxin polar transport, is essential for the asymmetric distribution of auxin and relies on three transport proteins: auxin-resistant (AUX/LAX), PIN-FORMED (PIN), and ABCB/MDR/PGP family proteins. By regulating these proteins, plants can control the polar transport and distribution of auxin [24]. 

Previous studies have shown that indole-3-acetic acid positively regulates internode elongation in upland cotton [25]. In the case of *Pisum sativa*, a transcriptome study revealed that the lkb mutant, known for its shorter internodes, exhibited significantly lower levels of free IAA compared to the wild type [26]. Furthermore, experiments conducted on dark-and light-grown pea seedlings indicated a direct correlation between the quantity of free IAA and the extent of internode elongation. These findings suggest that the inhibition of stem growth controlled by phytochrome can be attributed to a decrease in free IAA levels [27]. Similar findings were obtained for Trifoliate Orange (*Poncirus trifoliata* L.), Moso Bamboo (*Phyllostachys edulis*), and rice, where IAA was identified as the key hormone positively regulating plant internode elongation [28,29,30].

In our study, we observed decreased levels of IAA in short-stalk castor plants, which was consistent with the expression of certain genes involved in the auxin signal pathway and auxin transport pathway, including *auxin-induced protein AUX22* (*LOC8271341*), *auxin-responsive protein IAA33* (*LOC8289729*), *auxin-responsive protein SAUR* (*LOC107260773*, *LOC8259049*, *LOC8260421*), and *ABC transporter G family member* (*LOC8277074*, *LOC8282982*, *LOC82985*). Disruptions in auxin biosynthesis, transportation, or signal transduction generally result in the suppression of apical growth, leading to increased branch growth and development. This ultimately leads to a dwarf phenotype and high yield [31,32,33]. Mutations in *TIR1* (*transport inhibitor response1*) disrupt the IAA signal network and decrease the content of IAA, leading to a dwarf phenotype of *Arabidopsis* [34]. Similar dwarfism can be observed for *Arabidopsis* and rice [35,36]. 

**Figure 10:**
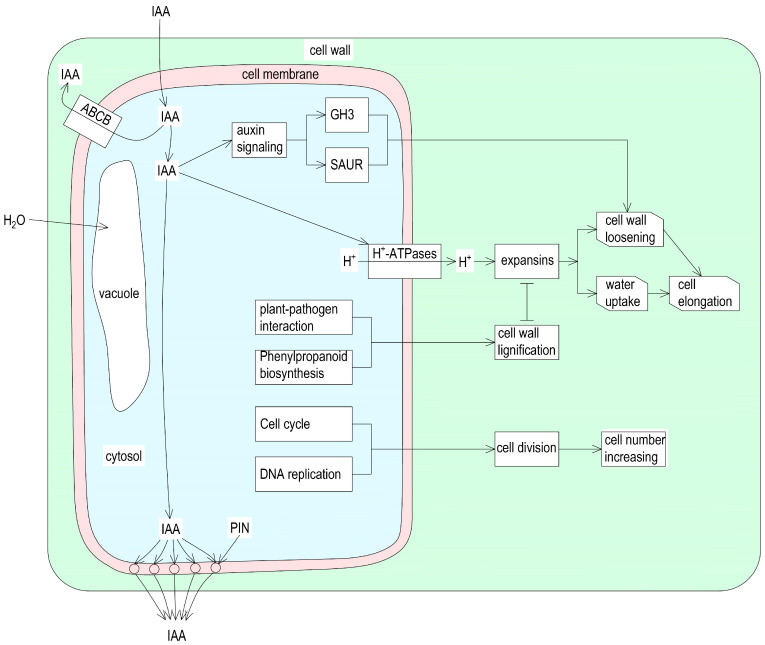
The model of cell elongation in castor internode tussue.

Our research demonstrates that polar auxin transport is responsible for the disparity in castor internode elongation and growth. We developed a model of cell elongation in castor bean internodes (Figure 10), which proposed that the variation in the elongation of castor bean internode tissues could be attributed to polar auxin transport. Auxin, synthesized in the shoot tips and young leaves, is transported downward through polar transport [24]. Upon entering the cytosol, IAA activates H^+^-ATPases, leading to ATP catabolism and the production of H^+^ ions. These H^+^ ions are then released into the cell wall region, resulting in a decrease in pH and the acidification of the cell wall area. In the acidic environment, expansin proteins catalyze interactions among cell wall components, weakening the cell wall and causing it to relax. As a result, the intracellular water potential decreases and extracellular water flows into the cell, causing an expansion of the cell volume. This process aligns with the acid growth theory and promotes growth extension [37,38]. Hence, the gene expression patterns of differentially expressed genes associated with plant hormones, cell division, and DNA replication, along with the variations in indole-3-acetic acid (IAA) content, provide substantial evidence in support of our proposed model. Our transcriptomic analysis demonstrated that dwarf castor bean plants exhibited decreased activity in the processes of cell cycle, cell division, and cell elongation, as compared to the internode cells of high-stalk plants.

### 3.2. Secondary Cell Wall Lignification Hinders Internode Elongation

Furthermore, certain key enzymes in the phenylpropane metabolism pathway, which is responsible for lignin synthesis, promote the lignification of the cell wall. This lignification prevents the cells from extending and ultimately leads to stunting. During the increase in cell number, cell division-related genes and DNA replication-related genes play a crucial role.

A histological analysis revealed that the stem of dwarf 071113 exhibited more advanced vascular tissue development. Furthermore, the vascular tissue of the dwarf 071113 castor plant displayed pronounced lignification in the transverse section of the fifth main stem internode. This finding aligns with the established notion that polar auxin transport can induce the development of vascular tissue. Interestingly, the high-stalk castor plant exhibited a higher auxin content compared to the short-stalk castor plant. This higher auxin content was found to be more conducive to vascular tissue development. In contrast, the low auxin content observed in the short-stalk castor plant induced the lignification of vascular tissue and impeded its development. Remarkably, these findings mirror the early lignification of vascular tissue observed in the *Arabidopsis pin-1* mutant [39,40]. Collectively, these results underscore the crucial role of polar auxin transport in vascular tissue development, while also highlighting the influence of auxin content and transport on plant height.

The metabolomic data successfully identified both the types and relative quantities of lignin small-molecule precursors present in short-stalk and high-stalk castor internode tissues. Lignin is primarily formed through the polymerization of three small molecular monomers: coniferyl alcohol (G monomer), sinapyl alcohol (S monomer), and p-coumaryl alcohol (H monomer). The contents of monomers G and S were significantly higher in the internode tissues of the dwarf castor plant, while the content of monomer H did not show significant differences. Furthermore, the precursors of the S monomer, sinapic acid and sinapinaldehyde, and the precursor of the H monomer, p-Coumaric acid, as well as the precursor of the G monomer, 4-Hydroxy-3-methoxycinnamaldehyde, were expressed at higher levels in the dwarf castor plant compared to the high-stalk castor plant. Additionally, certain key gene encoding enzymes involved in the lignin biosynthesis metabolic pathway showed up-regulated expressions in the short-stalk castor plant. This is similar to the findings for maize dwarf mutants, which display reduced stem elongation and identify several genes involved in secondary cell wall development. These genes include those responsible for cellulose synthesis (e.g., *CesA* genes) and lignin biosynthesis (e.g., *CCoAOMT* and *COMT* genes) [41]. Similar genes and pathways can also play a role in helping us to understand secondary cell wall development in castor bean internodes. The development and composition of secondary cell walls in plant tissues, including the castor bean main stem internode, are crucial for stem elongation and overall plant growth. Disruptions in secondary cell wall biosynthesis or an altered composition can result in stunted internodes and dwarfism.

Secondary growth involves the process of lignification, where lignin is deposited in the cell walls to provide mechanical strength and rigidity to plant stems [42]. This deposition is regulated by genetic and biochemical factors, with transcription factors, such as MYB and NAC, playing a crucial role in activating the genes responsible for lignin biosynthesis enzymes in castor stem transcriptomes. These enzymes include *phenylalanine ammonia-lyase* (*PAL*), *cinnamyl alcohol dehydrogenase* (*CAD*), and *peroxidases*. It is worth noting that internode elongation and secondary growth processes are interconnected. As internode elongation increases, the stem diameter also increases, creating a suitable environment for secondary growth to occur. This interdependence ensures the coordinated and efficient development of stems so they can fulfill their structural and functional roles in the plant.

## 4. Materials and Methods

### 4.1. Plant Materials

The castor bean (*Ricinus communis* L.) variety Zhuansihao was developed by cross-breeding the Lm-type female line with the Zhebisi variety. This strain is characterized by its compact plant structure, 4–5 main stem branches, thicker stalks, and a height of 280–300 cm. The stalk internodes develop normally, making it a representative high-stalk castor strain. On the other hand, the dwarf 071113 variety is derived from the crossbreeding of the dwarf castor variety with the Zhebisi castor bean variety. This selection line exhibits a relatively compact plant structure, with the main stem having 3–4 branches. The height of the 071113 strain is shorter, ranging from 130–150 cm, and it features a thicker stem and shortened internodes. Additionally, it forms tight clusters. These characteristics make the 071113 strain a typical dwarf castor variety. 

The Zhuansihao and 071113 castor varieties were cultivated in the fields of the Tongliao Agricultural Science Research Institute in Inner Mongolia. These two varieties were selected due to their consistent growth patterns. Plant samples were taken from the 5th internode tissue of plants during the rapid growth period (8–10 leaves) for various analyses, including a cytological observation, hormone content, lignin and cellulose content determinations, metabolome sequencing, and transcriptome sequencing. Each sample was collected with three biological replicates.

### 4.2. Tissue Fixation, Staining, and Microscopy

The samples were collected from 2 varieties, dwarf 071113 and high-stalk Zhuansihao, by obtaining a 0.5 cm thick section from the 5th internode of the main stem. The samples were fixed in 50% FAA fixative and then dehydrated using a series of alcohol solutions with varying concentrations: 75% alcohol for 4 h, 85% alcohol for 2 h, 90% alcohol for 1.5 h, 95% alcohol for 1 h, anhydrous ethanol for 0.5 h, and anhydrous ethanol II for 0.5 h. To achieve transparency, the samples were treated with anhydrous ethanol mixed with Xylene (1:1) for 10 min, followed by Xylene I for 10 min, and finally Xylene II for 7 min. Then, the samples were embedded in paraffin wax and sliced. The sliced samples were then dewaxed using a sequence of solutions. The samples were stained and then subjected to differentiation and decolorization processes using toluidine blue O (0.5% [*w*/*v*] toluidine blue OCI 52040 in 2.5% [*w*/*v*] Na-carbonate, pH 11) for visualization under an Olympus model BX53 microscope (Olympus, Tokyo, Japan) [43]. The widths of the xylem and cortex, number of vascular bundles, and length of cortical cells were measured in both transverse and longitudinal sections of the main stem.

### 4.3. Plant Hormone Measurement

To analyze the phytohormone contents of IAA and cytokinin, an LC-MS/MS analysis was conducted on three biological replicates of two varieties (Zhuansihao, named L1–L3 for the high-stalk variety, and D1–D3 for the short-stalk variety). The samples (50 mg) were ground into powder and extracted with 0.5 mL of methanol/water/formic acid (15:4:1, *v*/*v*/*v*) at 4 °C. The extract was vortexed (10 min) and centrifuged at 14,000 rpm under 4 °C for 5 min. The supernatants were collected, vortexed (5 min),and centrifuged (5 min). The combined extracts were evaporated to dryness under a nitrogen gas stream, reconstituted in 80% methanol (*v*/*v*), ultraphoniced (1 min), and filtrated (PTFE, 0.22 μm; Anpel) before the LC-MS/MS analysis. The sample extracts were analyzed using an LC-ESI-MS/MS system (HPLC, Shim-pack UFLC SHIMADZU CBM30A system, https://www.shimadzu.com.cn/; MS, Applied Biosystems6500 Triple Quadrupole, Waltham, MA, USA). The analytical conditions were as follows: HPLC: column, Waters ACQUITY UPLC HSS T3 C18 (1.8 µm, 2.1 mm × 100 mm); solvent system, water (0.04% acetic acid): acetonitrile (0.04% acetic acid); gradient program, 90:100 *v*/*v* at 0 min, 40:60 *v*/*v* at 5 min, 40:60 *v*/*v* at 7 min, 90:10 *v*/*v* at 7 min, 90:10 *v*/*v* at 10 min, flow rate of 0.35 mL/min; temperature of 40 °C; and injection volume of 2 μL. The effluent was alternatively connected to an ESI-triple quadrupole-linear ion trap (Q TRAP)-MS. 

The mass spectrometry data were qualitatively analyzed using the database provided by Metware Biotechnology Inc.(Wuhan, Chian) (https://www.metwarebio.com) and processed using Analyst 1.6.1 software (AB SCIEX, Redwood, ON, Canada). The qualitative and quantitative determinations of the sample hormones were based on the chromatographic peak area, and a standard curve was created to calculate the absolute content of hormones. The hormone content (ng/g) was calculated using the following formula: hormone content (ng/g) = BC/1000/D, where B represents the concentration level (ng/mL) obtained by substituting the integrated peak area of the hormone in the sample into the standard curve, C represents the volume of the solution used for reconstitution (100 μL), and D represents the weight of the sample weighed (g). However, the hormone content of the L1 sample was identified as an outlier and was therefore substituted with the average value of the L2 and L3 sample data for the statistical analysis.

### 4.4. Lignin and Cellulose Measurements

The internode tissues of castor plants were rinsed with a PBS buffer solution for three biological replicates. The sliced tissue was then homogenized with PBS and centrifuged. The lignin and cellulose contents were determined through the employment of the lignin enzyme-linked immunosorbent assay kit (Shanghai Jianglai industrial Limited By Share Ltd., Shanghai, China) and the cellulose enzyme-linked immunosorbent assay kit (Shanghai Jianglai industrial Limited By Share Ltd., Shanghai, China), correspondingly. The measurements were performed at room temperature following the instructions of the kit. Samples were added to blank and sample wells, followed by the addition of the enzyme-labeled reagent. The mixture was then incubated for 1 h at 37 °C. After the incubation, the liquid was discarded and the wash step was repeated 5 times. The experiment involved the addition of color reagents A and B followed by 15 min of incubation in darkness. The reaction was terminated by the inclusion of the stop solution. The OD at 450 nm was subsequently measured employing the Multi-Mode Microplate Reader FlexStation 3 (Molecular Devices, San Jose, CA, USA).

### 4.5. Metabolome Comparative Analysis

Three biological replicates were obtained from the middle section of the 5th internode of the main stems of Zhuansihao and 071113 varieties. The metabolome data were analyzed by LC-MS/MS at Metware Biotechnology Inc. The data quality was evaluated and a principal component analysis was conducted. PCCs were also computed. D2 samples in group D were excluded from the analysis due to its poor reproducibility. The data for group D were based on D1 and D3 samples, and the data for group L were based on L1, L2, and L3 samples. DMs were identified using an FC ≥ 1.5 or ≤0.5 in D/L, and VIP ≥ 1. VIP ≥ 1 indicated significant differences and helped classify and differentiate samples within each group. 

The KEGG enrichment analysis was performed on the differential metabolites in the internode tissues of the two castor varieties using the KEGG pathway database (https://www.kegg.jp/kegg/pathway.html). The metabolite names were matched with the KO numbers in the KEGG database. The hypergeometric test method in the R package (https://www.r-project.org) clusterProfiler (v3.10.1) [44] was utilized for the enrichment analysis of the differential metabolites. After annotating the differential genes in the KEGG pathway, the up-regulation and down-regulation of the KO nodes containing these genes were indicated in the pathway diagram for a visual representation. Differentially up-regulated genes were indicated in red, while down-regulated differential genes were indicated in green. Nodes that contained both up-regulated and down-regulated genes were denoted in blue. 

### 4.6. Transcriptome Comparative Analysis

Transcriptome data acquisition, quality assessment, and sample correlation analysis.

Three biological replicates were collected from the middle sections of the 5th internodes of the main stems of the Zhuansihao and 071113 varieties to perform RNA-Sequencing at Metware Biotechnology Inc. The construction of the cDNA library involved the attaching of Oligo (dT) magnetic beads to the polyA tail of mRNA, reverse transcription to generate the first cDNA strand, followed by the synthesis of the second cDNA strand. The purified cDNA was then end-repaired and an A-tail was added before ligating to a sequencing adapter. To select for cDNA fragments of approximately 200 bp, AMPure XP beads were utilized. The final library was constructed through PCR amplification and subsequent purification. The library was sequenced on the Illumina HiSeq platform after a qualified quality inspection. Transcriptome data acquisition, quality assessment, and sample correlation analysis were performed. 

Based on the PCC results obtained from the analysis of samples within and between groups, certain criteria were applied to ensure consistency with the metabolome analysis. As a result, the D2 sample was excluded and only the D1 and D3 samples were considered as the two biological replicates for the D-group samples. Likewise, the L group consisted of the L1, L2, and L3 samples as biological replicates for the analysis of differentially expressed gene screening. DESeq2 was used to examine the differences in gene expressions between the sample groups, utilizing unstandardized read count data. The *p* values were adjusted through the Benjamini–Hochberg method to account for multiple hypothesis testing and to ascertain the false discovery rate (FDR). DEGs were classified based on the criteria of an absolute |log2FC| ≥ 2 for the D/L ratio and an FDR value of less than 0.05. The ggplot2 package (https://ggplot2.tidyverse.org) was employed to perform a expression cluster analysis and create a heatmap for the DEGs. The method of the KEGG enrichment analysis for DEGs was the same as that used for DM screening.

### 4.7. Real-Time qPCR

To verify the expression patterns observed in transcriptome sequencing, a total of seven DEGs were selected for validation using the RT-qPCR method. These DEGs were enriched in the pathways related to auxin signal transduction (*SAU50*, *LOC107260773*), cell wall biosynthesis (*PER*, *LOC8258115*; *PME45, LOC8288420*; *NAC*, *LOC8265938*; *PGLR*, *LOC107262217*), and protein processing in the endoplasmic reticulum (*HSP7C*, *LOC8265956*; *CLPB1*, *LOC8284441*). The primer design (Table 2) for RT-qPCR was created using Premier Primer 3.0 software (https://primer3.org/) and synthesized by Sangon Biotech (https://www.sangon.com/) using RNA-Seq sequencing sample cDNA as a template. PCR reaction system (20 μL): 2 × SYBR qPCR Master Mix 10 μL, cDNA template 1 μL, forward and reverse primers (10 μmol/L) 0.4 μL each, ROXⅡ 0.4 μL, and RNase Free H_2_O_2_ 7.8 μL. Reaction program: 95 °C pre-denaturation for 1 min; denaturation at 95 °C for 20 s, annealing at 60 °C for 40 s and extension at 72 °C for 30 s, 40 cycles; and dissolution curve: 95 °C for 15 s, 60 °C for 60 s, and 95 °C for 15 s. A total of 3 biological replicates for each group, 4 technical replicates, and relative expression calculated using 2^−ΔΔCt^ method. RT-qPCR was conducted using the 2 × SYBR Green qPCR Master Mix (NovoStart, Cat#E099, Beijing, China) on a QuantStudio 6 Flex (ABI, Foster, CA, USA). The expression of *Actin* (*LOC8269707*) was used as an internal control for normalization. 

### 4.8. Correlation Analysis of Transcriptom and Metabolomes

We calculated the PCCs for the DEG and DM molecules. The correlation between genes and metabolites was investigated using a correlation coefficient threshold of 0.8 and a correlation *p* value of 0.05. Furthermore, we conducted a KEGG enrichment analysis using the same methodology as previously described. We analyzed DEGs and DMs by mapping them to the KEGG pathway database and identifying the up–down-regulation relationships of KO nodes. 

### 4.9. Data Analysis

A data analysis was conducted using R software (4.3.2). The independent sample *t*-test method was employed. A statistically significant difference was identified when the *p* value was less than 0.05. Moreover, a highly significant difference was established when the *p* value was less than 0.01.

## 5. Conclusions

The height of the castor bean plant was significantly affected by the length of internodes on its main stem. During the rapid growth period, the internodes of the dwarf 071113 cultivar exhibited lower growth rates, a larger cortex width, a shorter length of cortical cells, more lignified vascular systems and secondary structures, lower levels of indole-3-acetic acid (IAA), and a higher lignin content compared to the high-stalk control. Additionally, both the transcriptome and metabolome analyses revealed that DEGs were enriched in pathways related to the cell cycle, plant hormone signal transduction, and phenylpropanoid biosynthesis. More research is needed to fully comprehend the specific mechanism of dwarfing in castor plants. By exploring the genetic, hormonal, and physiological aspects of internode development in castor beans, we can gain insights into the underlying mechanisms responsible for dwarfism in this species. Our research aims to establish a solid foundation for breeding short-stalk castor varieties, which will ultimately increase the crop yield and income in the future.

## Figures and Tables

**Table 1:** Differential metabolites between high-stalk and dwarf castor plants.

#ID	Name	log2FC	*p* Value	VIP	Regulated
pma0294	Chrysoeriol 5-O-hexoside	1.77	0.01	1.59	up
pma1751	Nicotinic acid-hexoside	0.90	0.03	1.53	up
pmb0542	Cyanidin 3-O-malonylhexoside	0.62	0.03	1.45	up
pmb0576	Apigenin O-malonylhexoside	1.27	0.03	1.52	up
pmb0607	Chrysoeriol 7-O-hexoside	1.58	0.03	1.62	up
pmb0608	Chrysoeriol O-malonylhexoside	1.79	0.04	1.59	up
pmb0852	LysoPC 18:2	1.20	0.03	1.47	up
pmb1240	Phellodensin F	0.61	0.05	1.45	up
pmb2406	LysoPC 17:0	0.76	0.04	1.43	up
pmb2556	Syringaldehyde O-glucoside	−0.71	0.01	1.53	down
pmb2601	7-hydroxycoumarin-beta-rhamnoside	−1.13	0.02	1.52	down
pmb2685	2-Deoxyribose 5′-phosphate	0.83	0.02	1.50	up
pmb2804	13-HPODE	0.70	0.03	1.49	up
pmb3023	Eriodictyol C-hexoside	−1.41	0.04	1.42	down
pmb3026	Quercetin O-acetylhexoside	0.74	0.05	1.61	up
pmb3028	Tricin O-sinapic acid	1.13	0.01	1.57	up
pme0208	Amentoflavone	1.48	0.00	1.62	up
pme0250	Azelaic acid	0.64	0.02	1.51	up
pme0398	Chlorogenic acid (3-O-Caffeoylquinic acid)	−1.19	0.01	1.55	down
pme0413	Vanillin	0.82	0.05	1.42	up
pme0534	Gluconic acid	1.16	0.00	1.60	up
pme0543	Indole-5-carboxylic acid	0.64	0.03	1.50	up
pme1521	Dihydroquercetin (Taxifolin)	0.62	0.02	1.54	up
pme1583	Eriodictyol	−1.13	0.01	1.59	down
pme1622	Kaempferol 3-O-glucoside (Astragalin)	−0.66	0.01	1.56	down
pme1637	Coniferyl alcohol	1.17	0.04	1.43	up
pme2054	L-Tryptophan	−1.08	0.03	1.52	down
pme2901	1-O-Caffeoyl quinic acid	−1.59	0.01	1.60	down
pme2957	Naringenin chalcone	−0.73	0.03	1.50	down
pme2984	Isosakuranetin-7-neohesperidoside (Poncirin)	−1.59	0.02	1.55	down
pme3123	Sinapyl alcohol	1.22	0.00	1.60	up
pme3191	Uridine 5′-monophosphate	−0.87	0.04	1.45	down
pme3210	Genistein 7-O-Glucoside (Genistin)	1.39	0.03	1.58	up
pme3246	Coniferin	−0.60	0.03	1.50	down
pme3396	Fustin	−1.02	0.00	1.63	down
pme3431	“Esculin (6,7-Dihydroxycoumarin-6-glucoside)”	−1.01	0.02	1.51	down
pme3443	Sinapinaldehyde	1.48	0.00	1.58	up
pmf0057	“4,2′,4′,6′-Tetrahydroxychalcone”	−0.74	0.00	1.61	down
pmf0232	Tiliroside	−1.62	0.01	1.55	down
pmf0348	“2,6-Dimethyl-7-octene-2,3,6-triol”	0.65	0.02	1.53	up
pmf0455	Peimine	2.32	0.00	1.63	up
pmf0559	Abrine	1.16	0.02	1.56	up

**Table 2:** Primer sequences.

Genes	Primer Sequences	
*LOC107262217 (PGLR)*	F: GACCTGGACATGGCATTAGTATAG	R: CCATTAGCGGTGTTTGAGAGA
*LOC8265938 (NAC)*	F: CTATAGCCGGAGAACACAGAATAG	R: CAACTCTCTTGTTGGCGTTTG
*LOC8265956 (HSP7C)*	F: ATACCACCATTCCCACAAAGA	R: CTTGCTCTCTCACCCTCATAAA
*LOC8284441 (CLPB1)*	F: CACTGATGCTGCATTGGATTAC	R: TAACTGTGTCACCACCTTCTTC
*LOC107260773 (SAU50)*	F: GCCGAAGAAGAGTTTGGATTTG	R: GTGACGATGTTAGAGAGCGAAA
*LOC8288420 (PME45)*	F: CCCTCATTGGTGGCTTCATC	R: CTTACAGCCTGCAATCCACC
*LOC8258115 (PER)*	F: ATTCTCGCCGCCTTCTACAG	R: GTGGGAGCTTGGCATTGTTG
*LOC8269707 (Actin)*	F: AGGAGTTGGGTGTGTTCATTC	R: ACAAGGACTCCACAGCTTTATC

## Data Availability

The transcriptome datasets for this study are available from the GEO databank, accessible at https://www.ncbi.nlm.nih.gov/geo. The corresponding accession number is GSE242985. To review GEO accession GSE242985, please visit https://www.ncbi.nlm.nih.gov/geo/query/acc.cgi?acc=GSE242985 (accessed on 10 January 2024) and enter the token mzmjmey-iltqnhkp into the box. The metabolomic data can be accessed at www.ebi.ac.uk/metabolights/ MTBLS9036.
